# Diet‐Driven Divergence in Gut Microbiota Variation Between Two Sympatric Gerbil Species

**DOI:** 10.1002/ece3.73367

**Published:** 2026-04-17

**Authors:** Dongyang Chu, Nan Liu, Qingxuan Liu, Xin Li, Haizhou Yang, Na Zhu, Zhiying Liu, Rui Wang, Shuai Yuan, Heping Fu

**Affiliations:** ^1^ College of Grassland Science, Inner Mongolia Agricultural University, Inner Mongolia Agricultural University Hohhot China; ^2^ Alxa Left Banner Field Scientific Observation and Research Station for Desert Ecology and Rodent Control, Inner Mongolia Autonomous Region Hohhot China; ^3^ East Ujimqin Banner Field Scientific Observation and Research Station for Typical Grassland Pest Control, Inner Mongolia Autonomous Region Hohhot China

**Keywords:** dietary habits, gerbils, gut microbiome, sympatric coexistence

## Abstract

Gut microbiota provide various benefits to their mammalian hosts; however, knowledge regarding interspecific differences in gut microecology remains limited. This study employed 16S rRNA sequencing combined with metagenomic functional prediction (potential functions or functional potential) to conduct a comparative analysis of the gut microbial composition and functional adaptability of two sympatrically distributed gerbil species with distinct diets: the herbivorous *Rhombomys opimus* (RO) and the omnivorous *Meriones meridianus* (MM). The results revealed that the omnivorous MM exhibited a level of gut microbial alpha diversity comparable to that of the herbivorous RO, whereas RO showed significant enrichment of *norank_f__Muribaculaceae*, a taxon associated with fiber degradation, and demonstrated higher abundance of genes related to complex fiber degradation. Notably, bacterial genera significantly enriched in the gut of MM, such as *Lachnospiraceae_NK4A136_group* and *Desulfovibrio*, may play important roles in maintaining gut health and enhancing chitin degradation efficiency. Furthermore, the abundance of genes related to monosaccharide and chitin degradation was significantly higher in MM than in RO. Functional network analysis indicated that the cellulose degradation gene networks in both gerbil species were predominantly synergistic, but the synergistic effect was stronger in RO than in MM (ratios of positive to negative correlation edges: 2.44: 1.59). Further analysis revealed that the monosaccharide and chitin degradation gene networks in MM both exhibited synergistic interaction patterns (ratios of positive to negative correlation edges: 1.69 and 2.95, respectively), whereas these two networks in RO were primarily antagonistic (ratios of positive to negative correlation edges: 0.831 and 0.73, respectively). This suggests that the gut microbiota of RO are more conducive to digesting complex plant fibers, while those of MM are better adapted for digesting starch and chitin. This differentiation in gut microbiota optimizes the utilization of different food resources by the two species, thereby promoting their sympatric coexistence. This study enhances our understanding of the adaptive mechanisms of gut microecology in rodents with different diets and provides an important foundation for further research on the microbial ecology of wild rodents and the mechanisms underlying sympatric species coexistence.

## Abstract 2

This study compared the gut microbiota of sympatric herbivorous ( *Rhombomys opimus* ) and omnivorous ( *Meriones meridianus* ) gerbils. Results showed that the omnivorous species had higher microbial diversity and enriched genes for monosaccharide and chitin degradation, with cooperative functional networks, while the herbivorous species specialized in fiber‐degrading taxa and genes but exhibited antagonistic networks for other nutrient processing. These findings reveal diet‐driven adaptations in gut microecology and provide insights into wild rodent microbial ecology.

## 1 Introduction

The gut microbiome of mammals constitutes a highly complex and biodiverse ecosystem, with the total number of genes it encodes estimated to be approximately 150 times greater than that of the host's own genome (Qin et al. 2010), and is therefore often referred to as the host's “second genome.” The structure and function of this microbial community are governed by a range of multidimensional factors. These include intrinsic determinants, such as the host's genetic background, taxonomy, and developmental stage, as well as extrinsic influences like dietary composition, geographic habitat, and social behavior (Amato et al. 2013). Among these numerous factors, host phylogenetic relationships and dietary strategies are widely recognized as two principal driving forces that shape the composition and functional evolution of the gut microbiota (Mallott and Amato 2021). Phylogenetic relationships capture the conservative selection of microbial communities over the course of the host's long‐term evolutionary history (Brooks et al. 2016), whereas diet exerts direct selective pressures through the daily intake of nutrients, thus driving the dynamic adaptation and functional differentiation of the microbial community (Ecklu‐Mensah et al. 2022).

The extensive metabolic diversity within gut microbial communities endows the host with a broad array of distinctive functions and directly contributes to its physiological activities and energy metabolism. Consequently, there is an increasing consensus that the microbiome may serve as a key mediator enabling the host to achieve rapid ecological adaptation and evolutionary diversification (Lindsay et al. 2020). Among these functions, a particularly important one is the microbial ability to degrade complex biopolymers. This process expands the range of nutrient resources available to the host by breaking down substances indigestible to the host itself. For instance, structural components of plant cell walls—such as cellulose and hemicellulose—are carbohydrate polymers. These polymers are composed of β‐1,4‐glycosidic bonds that cannot be hydrolysed by the host's endogenous enzymatic systems (Karasov and Martínez del Rio 2007). Thus, most herbivorous mammals depend on gut microbes to perform anaerobic fermentation, transforming these otherwise indigestible polysaccharides into absorbable metabolites, such as short‐chain fatty acids, which constitute important sources for maintaining the host's energy balance. Another biopolymer of notable significance is chitin. As the second most abundant polysaccharide in nature (Tharanathan and Kittur 2003)^,^ chitin is widely distributed in the exoskeletons of arthropods and the cell walls of fungi and is a linear polymer composed of N‐acetylglucosamine units linked by β‐1,4 bonds. It is a substantial dietary component for many insectivorous and omnivorous vertebrates. Research has shown that specialized mammals which feed predominantly on ants and termites—such as *Myrmecophaga tridactyla* , *Manis pentadactyla* , and *Orycteropus afer* (Teullet et al. 2023)—have evolved strikingly similar gut microbial communities despite being phylogenetically distant. In addition, studies have found significant differences in the relative abundance of gut bacteria and gene functions between geckos fed a chitin‐rich diet of mealworms and their wild conspecifics (Jiang et al. 2023). Together, these findings suggest that microorganisms may play a key role in facilitating host adaptation to chitin‐rich diets. However, little is currently known about the prevalence and functional significance of such microbial convergence among other insectivorous taxa (Delsuc et al. 2014). Key questions remain: whether these communities indeed increase chitin digestion efficiency, and which specific microbial taxa are involved and play critical roles in this process. In particular, for omnivorous species that are not yet specialized and that regularly ingest chitin, whether their microbiomes undergo similar adaptive modifications remains to be systematically investigated.

Rodents serve as excellent models for investigating fundamental questions in host–microbe interactions. As the most species‐rich order among mammals, they exhibit diverse ecological adaptations, occupy a broad range of habitats, and display notable dietary variation (Auffray et al. 2009). Consequently, rodents have become a widely utilized model system in comparative biology (Gorbunova et al. 2008), particularly within the realm of host–microbe interactions (Weinstein et al. 2021a). Both *Rhombomys opimus* and *Meriones meridianus* belong to the order Rodentia and the family Muridae and are commonly found in the desert regions of Central Asia. The former is the sole representative of the genus Rhombomys, whereas the latter is classified under the genus Meriones. Both species are widely distributed across northern China (Wei et al. 2025). Notably, in certain habitats, their ranges frequently overlap. In regions where RO is present, MM often occurs as a sympatric species, resulting in a stable pattern of coexistence (Zhao 2006). Research has indicated that this coexistence is largely facilitated by pronounced trophic niche differentiation: RO is a specialized herbivore with a restricted diet, feeding primarily on moisture‐rich green stems and leaves of plants; in contrast, MM is a typical omnivore, consuming plant matter alongside a substantial proportion of insects and seeds (Zhang 2004). This marked dietary divergence mitigates direct competition for limited resources and constitutes a key niche differentiation strategy that underpins their long‐term stable coexistence within overlapping distribution areas.

To determine whether the aforementioned dietary divergence is reflected in the gut microbiome structure, which is closely associated with digestive function, this study employed 16S rRNA gene high‐throughput sequencing to characterize the gut microbial communities of two sympatric gerbil species inhabiting the same region but adopting distinct feeding strategies. Predictive metagenomics (PICRUSt2) was further applied to infer their potential functional capacities. On the basis of the documented dietary differences between the two species, we formulated the following hypotheses. (i) The two gerbil species will display markedly distinct gut microbiome structures; (ii) The microbial communities, in terms of both taxonomic composition and predicted functions, will be enriched with microbial taxa and functional gene sets associated with the decomposition and metabolism of the principal components of their natural diets (e.g., plant cellulose for RO and chitin for MM).

## 2 Methods

### 2.1 Rodent Collection

**FIGURE 1:**
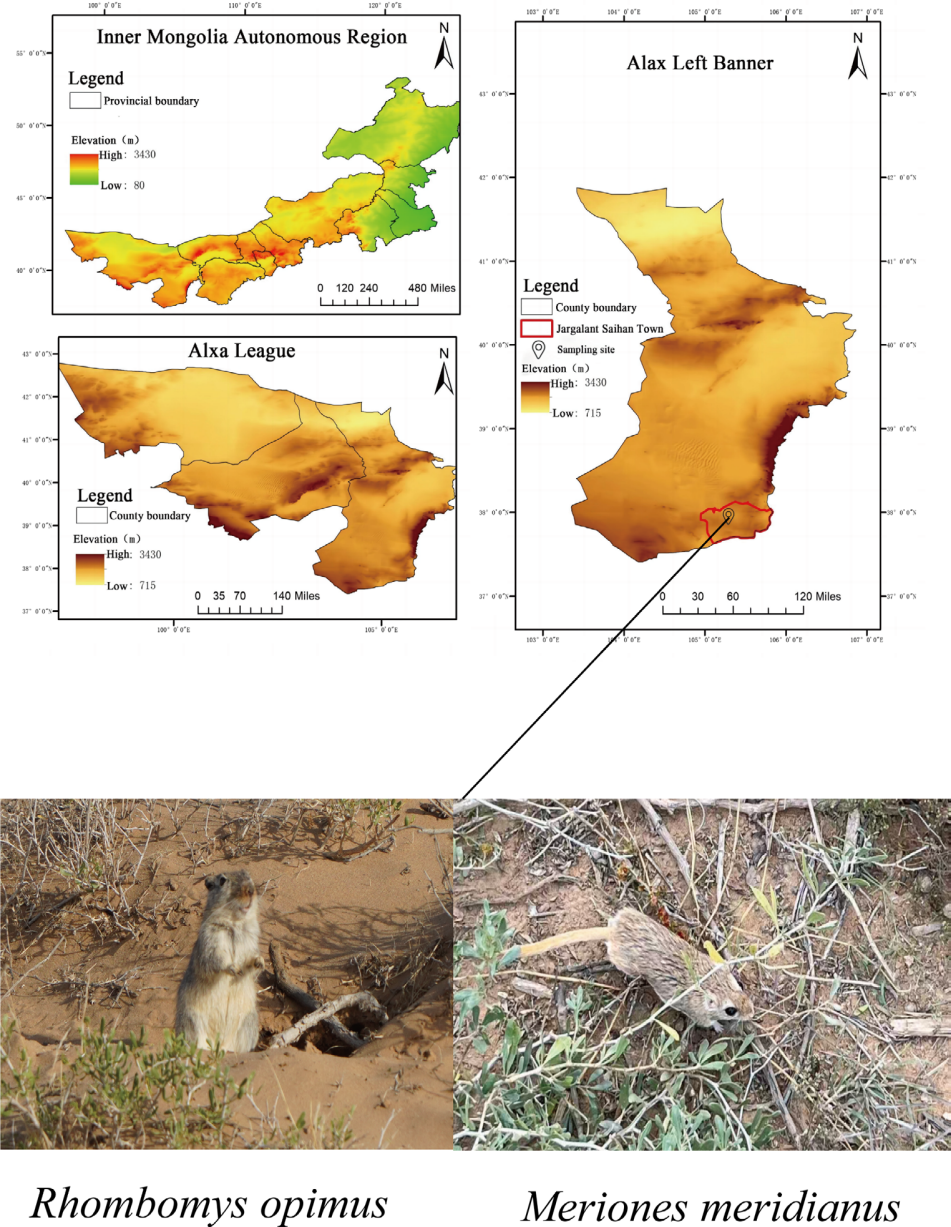
Sampling site and photographs of great gerbils and midday gerbils (Authors’ own).

In 2024, two rodent species were collected from the desert region in the southern part of Alxa Left Banner, Alxa League, Inner Mongolia Autonomous Region, China (37.887497° N, 105.381715° E) including 10 *Rhombomys opimus* and 10 *Meriones meridianus* , with five males and five females for each species (Figure 1, Table 1). Within the sampling area, live traps baited with fresh peanuts and carrots were employed to capture the rodents. The traps were set in the early morning and inspected in the evening, as well as the following morning. Previous research has indicated that short‐term bait consumption in live traps does not significantly influence the gut microbiome of rodents (McCleery et al. 2022).

**TABLE 1:** Characteristics of the study species.

Species	Taxonomic family	Group	Sample size (n) ♂: ♀	Average weight (g)	Diet
*Rhombomys opimus*	Muridae	RO	5: 5	164.5	Plant foliage (Zhao 2006)
*Meriones meridianus*	Muridae	MM	5: 5	62.3	Plant foliage, insects, seeds (Zhao 2006)

*Note:* Based on the dietary composition of the two gerbil species, we categorized them as herbivorous (RO) and omnivorous (MM), the same below.

Euthanasia of RO and MM was performed by experienced personnel using an inhalation overdose of isoflurane, with 3.5% isoflurane used for induction. After confirmation of unconsciousness, euthanasia was carried out by cervical dislocation. Following euthanasia, cecal content samples were collected from the rodents. All samples were obtained under sterile conditions, placed in 2 mL sterile cryotubes, promptly frozen in liquid nitrogen, and subsequently stored at −80°C in an ultra‐low temperature environment. This study was approved by the Ethics Committee of Inner Mongolia Agricultural University (Approval No. NND2017012).

### 2.2 DNA Extraction and 16S rRNA Sequencing

Total DNA was extracted from the cecal contents using the FastDNA Spin Kit for Soil, in accordance with the manufacturer's instructions. Cecal contents were selected because the caecum serves as the primary site of food fermentation in rodents and is characterized by high microbial density and activity (Yang et al. 2018). The extracted DNA was stored on dry ice and subsequently transported to Shanghai Majorbio Bio‐Pharm Technology Co. Ltd. The upstream primer 338F (ACTCCTACGGGAGGCAGCAG) and downstream primer 806R (GGACTACHVGGGTWTCTAAT) were used to amplify the V3–V4 region of the bacterial 16S rRNA gene via PCR (Ambardar et al. 2016), after which the resulting amplicons were pooled and purified. Library preparation was conducted using the NEXTFLEX Rapid DNA‐Seq Kit, and sequencing was performed to generate 2 × 300 base‐pair paired‐end reads. Detailed descriptions of the amplification, library preparation, and sequencing procedures are provided in the (File S1). Sequence reads have been deposited in the NCBI database under accession number (PRJNA1274967).

Quality control of the raw sequencing data was conducted using fastp (https://github.com/OpenGene/fastp, version 0.20.0) (Chen et al. 2018), whereas FLASH (http://www.cbcb.umd.edu/software/flash, version 1.2.7) was employed for read merging (Magoč and Salzberg 2011). Bases with quality scores below 20 at the ends of the reads were removed. A 50 bp sliding window was applied, and if the average quality within the window fell below 20, bases from the start of the window to the 3′ end were trimmed. Reads shorter than 50 bp after quality control, as well as those containing N bases, were discarded. Paired‐end reads were merged into single sequences on the basis of overlapping regions, requiring a minimum overlap of 10 bp. A maximum mismatch rate of 0.2 was permitted within the overlap region, and sequences not meeting these criteria were excluded. Samples were identified according to barcodes and primers at both ends of the sequences, with read orientations adjusted accordingly. Zero mismatches were allowed for the barcodes, and up to two mismatches were permitted for the primers. OTU clustering at 97% sequence similarity was performed using UPARSE (http://drive5.com/uparse/, version 7.1). Reads identified as originating from archaea, chloroplasts, or mitochondria were removed from further analysis (Stackebrandt and Goebel 1994). Taxonomic classification of each sequence was carried out using the RDP classifier (http://rdp.cme.msu.edu/, version 2.2) against the Silva 16S rRNA database (v138), with a confidence threshold of 70% (Wang et al. 2007).

### 2.3 Statistical Analysis

We used QIIME2 to generate microbial abundance tables at various taxonomic levels by collapsing the feature table according to taxonomy, clustered bacterial OTUs using the UPARSE algorithm with a 97% sequence similarity threshold. We performed statistical analyses of OTUs using Usearch software to determine the composition and quantity of shared and unique OTUs in the gut microbiota of RO and MM. To compare gut microbial community diversity between the two gerbil species, we first calculated alpha diversity indices of the gut microbial communities using Mothur software and then applied Kruskal–Wallis tests to assess differences in alpha diversity, followed by post hoc tests to further identify significant intergroup differences. To subsequently examine differences in the gut microbial community composition, we computed pairwise community dissimilarities for all the samples on the basis of the Bray–Curtis distance (Bray and Curtis 1957), unweighted UniFrac distance, and weighted UniFrac distance (Lozupone and Knight 2005) and compared the compositional characteristics of the gut microbiota between the two species. Concurrently, nonmetric multidimensional scaling (NMDS) was employed to construct a visual representation, providing an intuitive depiction of similarities and differences among gut microbial communities from different samples. To further validate the microbiome changes (including community dispersion and compositional differences), we adopted two analytical approaches: (1) the PERMDISP test (with 999 permutations) in QIIME2 to evaluate the homogeneity of samples within groups in relation to community dispersion and (2) the calculation of Bray–Curtis distances between paired samples within the same species (intraspecific), followed by Kruskal–Wallis tests with post hoc multiple comparisons to assess the extent of intraspecific community variation.

To assess the composition of the bacterial communities in the two gerbil groups, we employed the taxa‐barplot tool in QIIME2 to visualize taxonomic abundance. To evaluate differences in bacterial communities between the groups, we used the LEfSe function in STAMP software to identify bacterial taxa exhibiting significantly different abundances at the genus level. Prior to conducting the differential analysis, we excluded bacterial taxa whose read counts were less than 20 to eliminate rare taxa (Weinstein et al. 2021b), and the analysis was primarily performed using default parameters.

To explore the functional potential of the gut microbiota, we employed PICRUSt2 (Phylogenetic Investigation of Communities by Reconstruction of Unobserved States) (Douglas et al. 2020) to generate predicted metagenomic functional profiles from 16S rRNA sequencing data. PICRUSt2 infers the functional capacity of a microbial community by mapping operational taxonomic units (OTUs) to a reference genome database and predicting gene family abundances based on phylogenetic relationships. It is important to note that the resulting functional profiles represent predicted functional potentials rather than directly measured metagenomic content, serving as an informative proxy for hypothesis generation and comparative analysis. In this process, all OTUs with a nearest sequenced taxon index (NSTI) value exceeding 2.0 were excluded. By acknowledging the potential limitations inherent in predictive metagenomic analysis, we prioritized pre‐established hypotheses and concentrated on assessing the predicted abundance of functional categories associated with biopolymer degradation.

We linked the glycoside hydrolase (GH) subset from the Carbohydrate‐Active enZymes (CAZy) database with the KEGG Orthology (KO) database (Lombard et al. 2014) to identify metabolic pathways relevant to our functional predictions. We examined genes involved in the digestion of simple sugars, including α‐glucosidase (K01187), oligosaccharide‐1,6‐glucosidase (K01182), α‐amylase (K07405), maltose‐6′‐phosphate glucosidase (K01232), and α‐amylase (K01176). To assess the microbial capacity for metabolizing complex fibers, we also analyzed the predicted abundances of genes associated with complex fiber degradation, such as β‐glucosidase (K05349), endoglucanase (K01179), endo‐1,4‐β‐xylanase (K01181), β‐glucosidase (K05350), and xylan‐1,4‐β‐xylosidase (K01198) by KEGG Orthology. Furthermore, we compared the relative abundances of multiple KEGG Orthology genes linked to chitin degradation (Borrelli et al. 2017), including chitinase (K01183), chitosanase (K01233), phospho‐chitobiase (K01222), and chitin disaccharide deacetylase (K03478). The predicted abundances of these genes in the two gerbil groups were compared using the Kruskal–Wallis test, followed by post hoc analysis.

An interaction network map of bacterial OTUs (top 500 in abundance) from RO and MM associated with cellulose, monosaccharide, and chitin degradation genes was generated using the “psych” package in R v4.5.1 and Gephi v0.9.2 (Dalcin and Jackson 2018). The network topology features were subsequently calculated with Gephi v0.9.2.

## 3 Results

**FIGURE 2:**
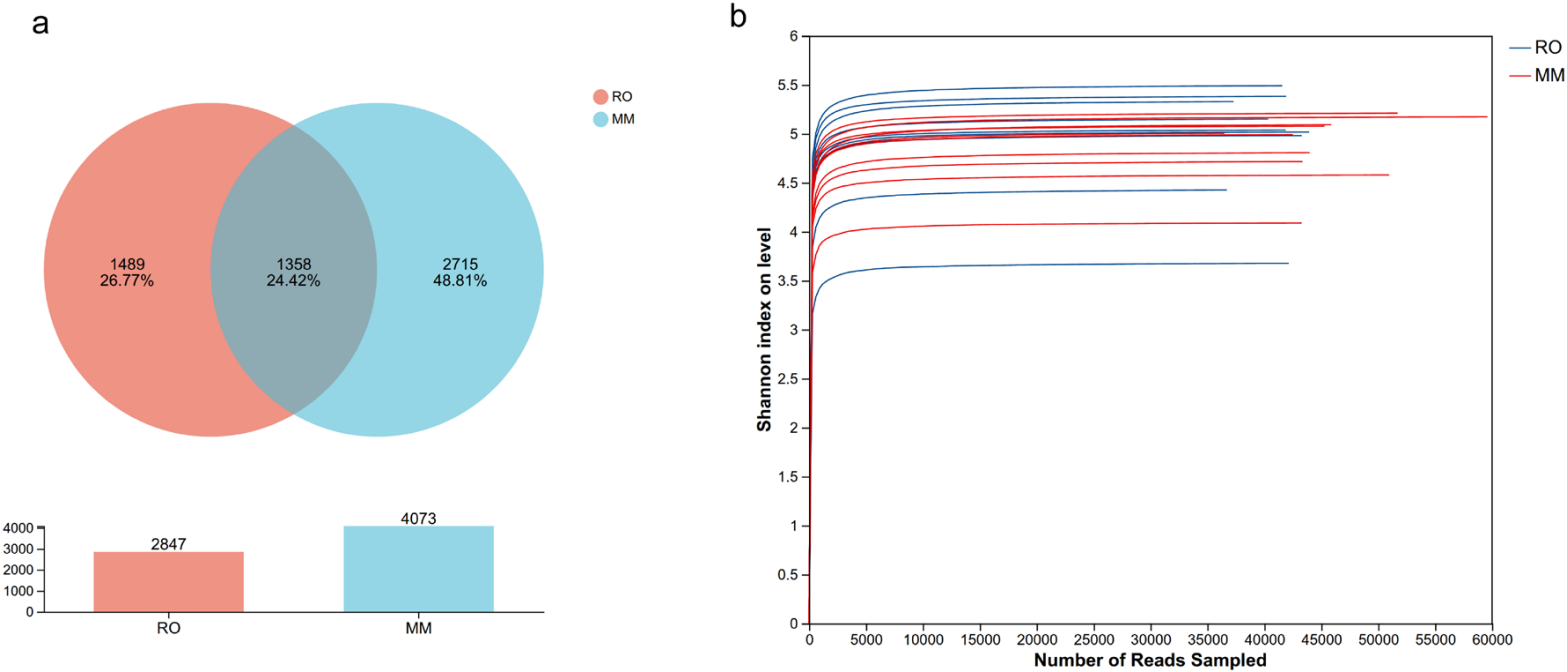
Sequencing quality and OTU analysis. (a) Venn diagram illustrating a comparison of the OTU counts between the two gerbil species. The two colored ellipses represent the respective species, and the numbers indicate OTUs unique to each species or shared by both; (b) Rarefaction curve of gut microbiota sequences.

This study examined the microbial composition of the cecal contents of two sympatric gerbil species. A total of 20 samples were analyzed, with the number of valid sequences per sample ranging from 29,592 to 36,472 and an average of 33,770 valid sequences. At the 97% sequence similarity threshold, 5562 core OTUs were identified. MM had 4073 shared OTUs and 2715 unique OTUs, whereas RO had 2747 shared OTUs and 1489 unique OTUs (Figure 2a). These findings suggest that the intestinal tracts of the two species contain a substantial proportion of shared bacterial taxa, along with distinct microbial communities unique to each species. Rarefaction curves Figure 2b indicated that the estimates of species richness approached a plateau and were unbiased, implying that the current sequencing depth adequately captured the vast majority of the bacterial diversity present in the samples, thus supporting its suitability for subsequent data analyses.

**FIGURE 3:**
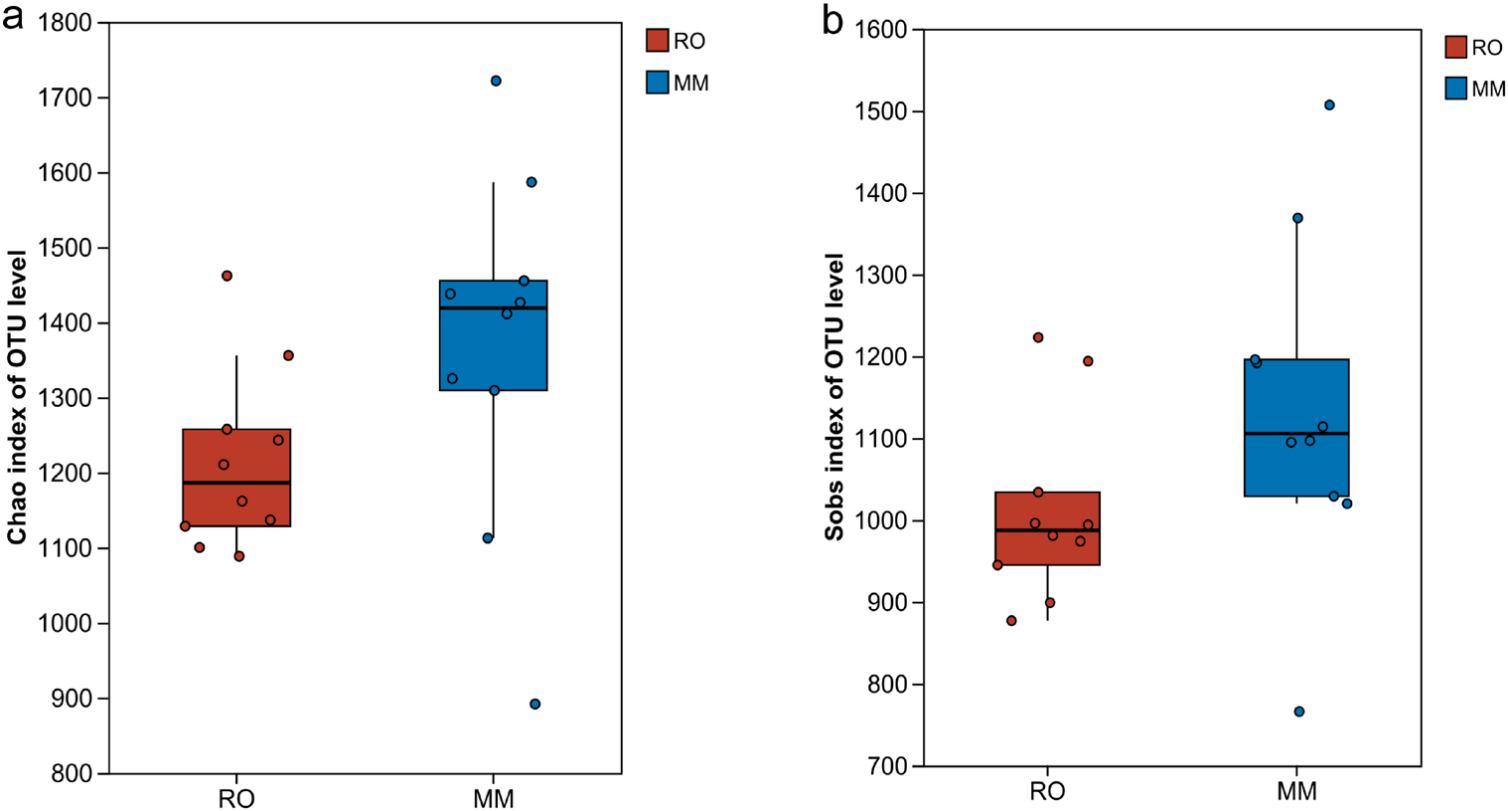
Variations in gut microbiota alpha diversity across different host species. (a) Chao diversity index; (b) Sobs diversity index.

We evaluated the alpha diversity of the gut microbial communities in two sympatric gerbil species using the Chao1 and Sobs indices to assess differences in species richness. The results showed that there was no significant difference in gut microbial alpha diversity between the omnivorous MM and the herbivorous RO (Chao1 index: *p* = 0.160; Sobs index*: p* = 0.248) (Figure 3a,b).

**FIGURE 4:**
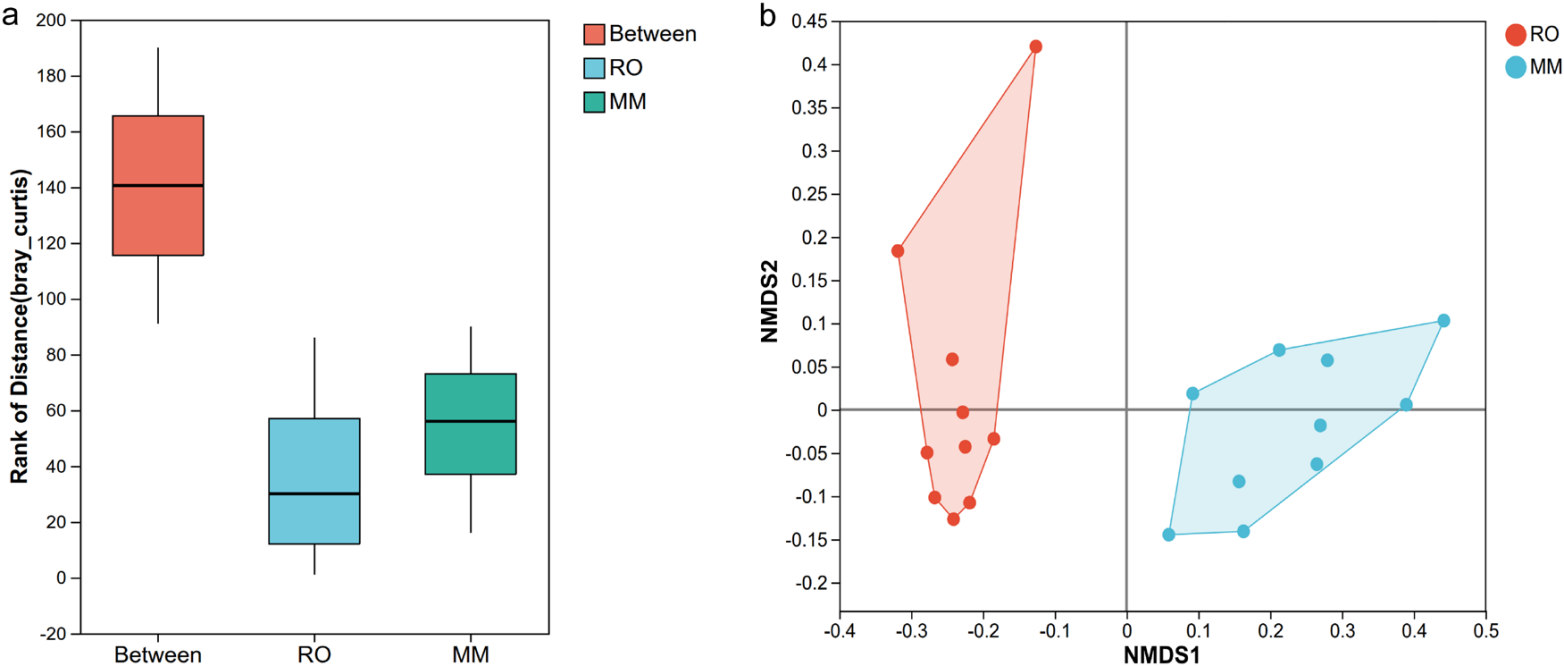
β diversity of the gut microbial communities of the two gerbil species. (a) In the ANOSIM box plot, the X‐axis denotes the grouping. The box labeled “Between” indicates the distance values reflecting intergroup differences, whereas the other boxes indicate the distance values for intragroup differences. The y‐axis scale reflects the magnitude of these distance values. The *R* value typically ranges from 0 to 1; the closer the *R* value is to 1, the greater the intergroup differences relative to intragroup differences. (b) NMDS analysis of the intestinal microbiota of two sympatrically coexisting gerbil species.

Individual rodents displayed distinct microbiota compositions. ANOSIM analysis was employed to assess intra‐ and intergroup differences between the two gerbil species, revealing that the omnivorous MM exhibited greater interindividual microbiota variation than the herbivorous RO did. Furthermore, interspecific differences were markedly greater than intraspecific differences (*R* = 0.6089, *p* = 0.001; Figure 4a). These findings were corroborated by NMDS analysis, which clearly revealed species‐level clustering of the microbiota in the two gerbil species (stress = 0.08, *p* = 0.001; Figure 4b). PERMANOVA analysis further confirmed that host species significantly influenced microbiome structure (*F* = 18.25, *p* = 0.001). Collectively, these results indicate that the gut microbiota structures of the two gerbil species differ substantially.

**FIGURE 5:**
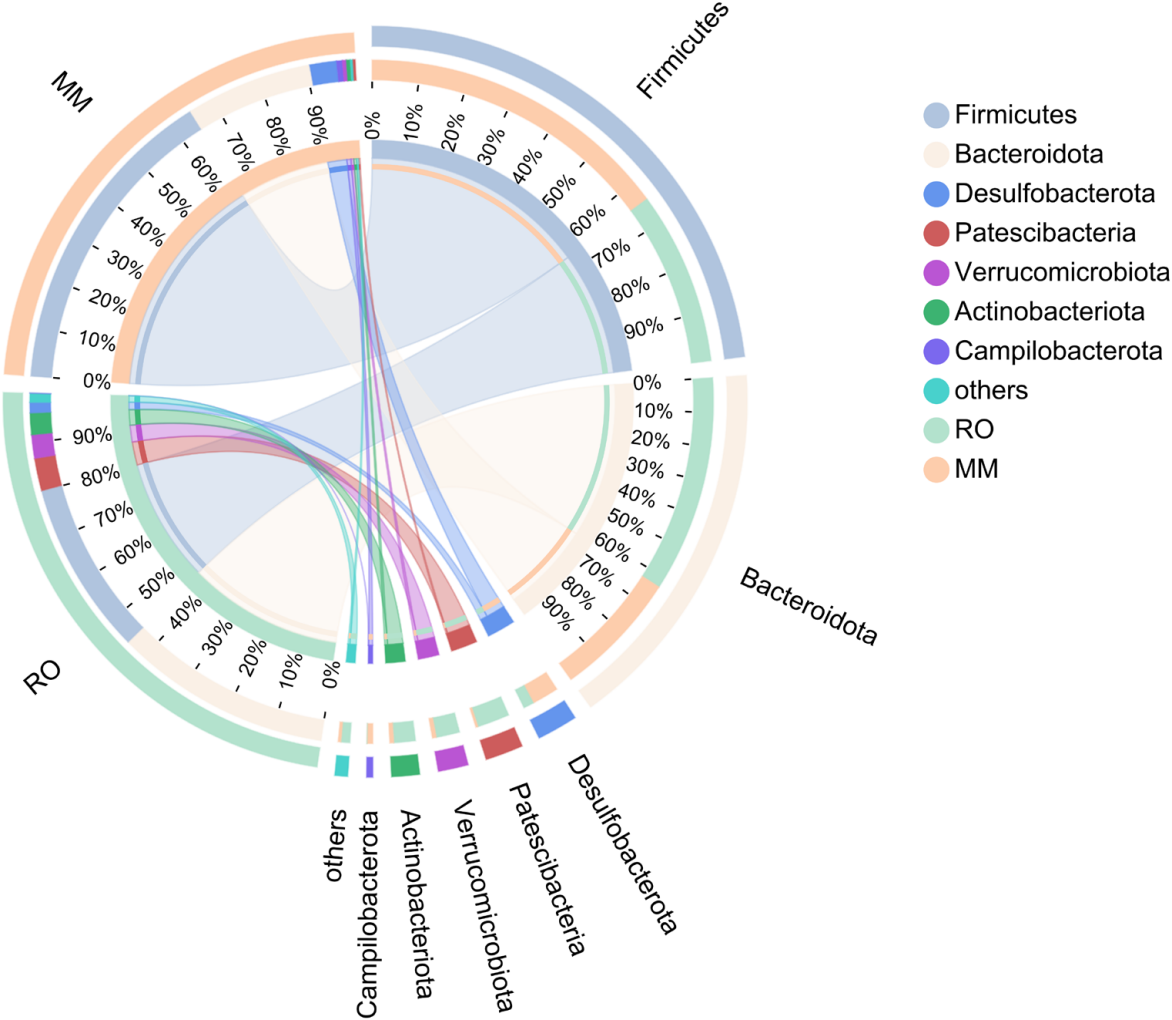
Circos plot depicts the compositional relationship between microbial taxa and sample groups. The left half of the circle represents species composition within samples: The color of the outer ribbon indicates the group of origin (MM or RO), with ribbon length corresponding to the total relative abundance of all species within that group; the color of the inner ribbon denotes specific microbial phyla (e.g., Firmicutes, Bacteroidota), and the width of each inner ribbon connection reflects the relative abundance of that phylum within the corresponding sample group. The right half of the circle displays the proportional distribution of each phylum across the two sample groups, where colored segments show, for a given phylum, what fraction of its total abundance is attributed to MM versus RO, revealing cross‐group distribution patterns.

We examined the bacterial composition of the gut microbiota of the two gerbil species at the phylum level and identified distinct abundance patterns. In MM, the gut microbiota was predominantly composed of Firmicutes (RO: 35%; MM: 65%), whereas in RO, Bacteroidota prevailed (RO: 63%; MM: 37%). Desulfobacterota and Campilobacterota were markedly enriched in the gut of the omnivorous MM. In contrast, Patescibacteria, Verrucomicrobiota, Actinobacteriota, Proteobacteria, Cyanobacteria, and Spirochaetota were more abundant in the gut of the herbivorous RO (Figure 5).

**FIGURE 6:**
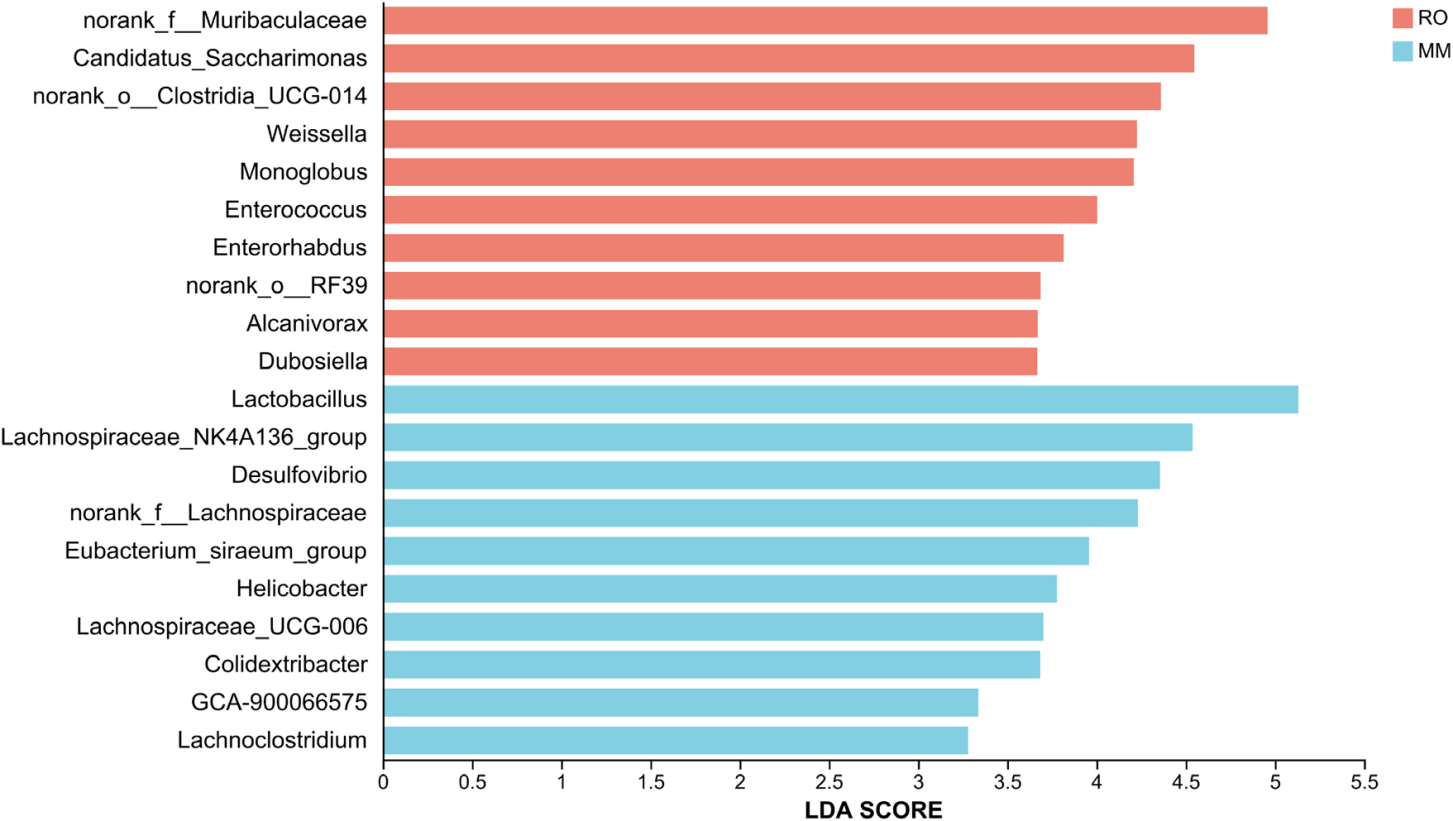
Linear discriminant analysis (LDA) scores of the gut microbiota at the genus level in both RO and MM.

At the genus level, we conducted a LefSe analysis (LDA > 3) on the gut microbiota of the two gerbil species. The results indicated that each species possessed its own uniquely enriched genera. In RO, the significantly enriched genera included *norank_f__Muribaculaceae* (specializing in the degradation of complex polysaccharides such as plant fibers) (Gan et al. 2023), *Weissella*, *Monoglobus*, *Candidatus_Saccharimonas*, *norank_o__Clostridia_UCG‐014*, *Enterococcus*, *norank_o__RF39*, *Alcanivorax*, and *Dubosiella*. In contrast, the gut microbiota of MM was significantly enriched in *Lactobacillus* (a genus of bacteria capable of lactic acid fermentation, converting carbohydrates into lactic acid) (Hynönen and Palva 2013), *Lachnospiraceae_NK4A136_group*, *Desulfovibrio* (a sulphate‐reducing bacterium and efficient hydrogen scavenger) (Fauque et al. 1988), *norank_f__Lachnospiraceae*, *Eubacterium_siraeum_group*, *Helicobacter*, *Lachnospiraceae_UCG‐006*, *Colidextribacter*, *GCA‐900066575*, and *Lachnoclostridium* (Figure 6).

When the microbial OTUs identified in our study were compared with the reference genomes in the PICRUSt2 database, the nearest sequenced taxon index (NSTI) for all 5562 OTUs was below the threshold of 2.0; consequently, none were excluded from the subsequent PICRUSt2 functional prediction analysis (File S2). The NSTI values across all the OTUs were generally low (mean ± standard deviation: 0.321 ± 0.194; median: 0.279). A significant difference in the weighted mean NSTI values was observed between the two gerbil species (Kruskal–Wallis test: *H* = 22.182, *p* < 0.001). The NSTI values obtained in this study are consistent with those reported in human microbiome research, suggesting that metagenomic functional predictions based on 16S rRNA gene sequencing data are reliable.

**FIGURE 7:**
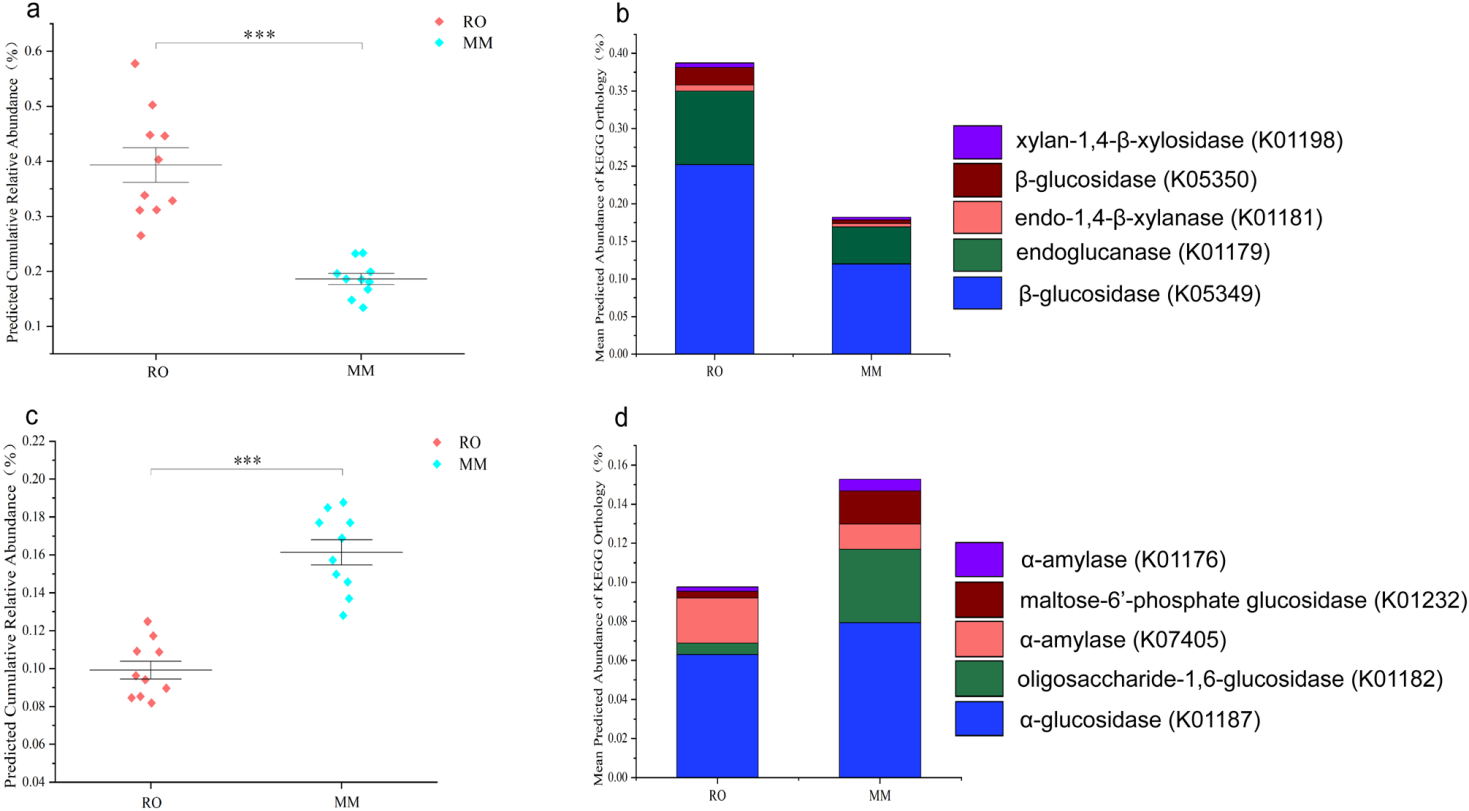
Predicted abundance of genes related to carbohydrate metabolism. (a) Predicted abundance of genes involved in the degradation of complex fibers. (b) Stacked bar chart illustrating the relative contributions of the five most abundant KEGG categories associated with fiber digestion. (c) Predicted abundance of genes involved in the degradation of monosaccharides. (d) Stacked bar chart illustrating the relative contributions of the five most abundant KEGG categories associated with monosaccharide digestion. In Panels (a) and (c), each dot represents data from an individual rodent, while the lines and bars denote the means ± standard errors. Brackets indicate the results of pairwise Kruskal–Wallis tests. Adjusted *p* values are denoted as follows: (***) for *p* < 0.001.

Functional prediction analysis revealed that the predicted abundance of fiber degradation‐related genes in the microbiota of the herbivorous RO was markedly greater, whereas it was substantially lower in the omnivorous MM (Figure 7a,b; Kruskal–Wallis test: *H* = 14.29, *p* < 0.001). Conversely, the predicted abundance of genes associated with monosaccharide digestion was greater in the omnivorous MM and lower in the herbivorous RO (Figure 7c,d; *H* = 14.29, *p* < 0.001). In both gerbil species, the most prevalent gene linked to complex fiber metabolism was β‐glucosidase (K05349) (Figure 7b), whereas the most prevalent gene associated with monosaccharide digestion was α‐glucosidase (K01187) (Figure 7d) (File S3).

**FIGURE 8:**
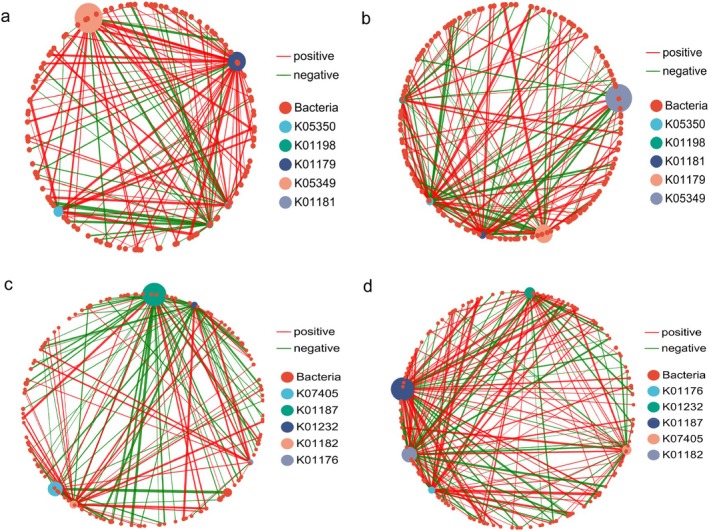
Interaction network maps illustrating the relationships between the top 500 most abundant bacterial OTUs of RO and MM and cellulose‐degrading genes (a and b), as well as monosaccharide‐degrading genes (c and d). Red lines denote positive associations, while green lines denote negative associations. Red nodes represent bacterial OTUs, whereas nodes in other colors correspond to distinct cellulose‐ and monosaccharide‐degrading genes. The relative abundances of these genes are reflected by the sizes of the dots.

In this study, Spearman correlation analyses were performed between the 500 most abundant bacterial OTUs in the intestinal tracts of the two gerbil species and genes associated with cellulose and monosaccharide degradation, respectively. In RO, 114 OTUs were significantly correlated with cellulose‐degrading genes (Table 2), whereas 126 OTUs were significantly correlated with monosaccharide‐degrading genes (Table 3). Within the cellulose‐degrading gene network of RO, 88 edges represented positive correlations (70.97%), and 36 represented negative correlations (29.03%) (Table 2, Figure 8a), yielding a positive‐to‐negative edge ratio of 2.44 and indicating a predominance of synergistic interactions. In the MM cellulose‐degrading gene network, 89 positively correlated edges (61.38%) and 56 negatively correlated edges (38.62%) were observed (Table 2, Figure 8b), resulting in a positive‐to‐negative edge ratio of 1.59, likewise indicating a predominance of synergistic interactions, although the degree of synergy was lower than that observed in RO. In the monosaccharide‐degrading gene network of RO, 59 positively correlated edges (45.38%) and 71 negatively correlated edges (54.62%) were identified (Table 3, Figure 8c), resulting in a positive‐to‐negative edge ratio of 0.831, suggesting that antagonistic interactions predominated between monosaccharide‐degrading genes and bacterial OTUs. Conversely, the monosaccharide‐degrading gene network of MM exhibited 110 positively correlated edges (62.86%) and 65 negatively correlated edges (37.14%) (Table 3, Figure 8d), corresponding to a positive‐to‐negative edge ratio of 1.69. This indicated that synergistic interactions predominated between monosaccharide‐degrading genes and bacterial OTUs in this species.

**TABLE 2:** Network topological characteristics of cellulose degradation.

	Cellulase genes network (RO)	Cellulase genes network (MM)
Nodes	114	145
Edges	124	145
Positive edges (%)	88 (70.97)	89 (61.38)
Negative edges (%)	36 (29.03)	56 (38.62)
Positive/Negative edges	2.44	1.59
Average degree	2.175	2
Diameter	6	4
Average path length	4.068	2.615
Density	0.019	0.014
Modularity	0.676	0.738

**TABLE 3:** Network topological characteristics of monosaccharide degradation.

	Monosaccharide catabolic genes network (RO)	Monosaccharide catabolic genes network (MM)
Nodes	126	134
Edges	130	175
Positive edges (%)	59 (45.38)	110 (62.86)
Negative edges (%)	71 (54.62)	65 (37.14)
Positive/Negative edges	0.831	1.69
Average degree	2.063	2.612
Diameter	8	6
Average path length	4.092	3.274
Density	0.017	0.02
Modularity	0.702	0.521

**FIGURE 9:**
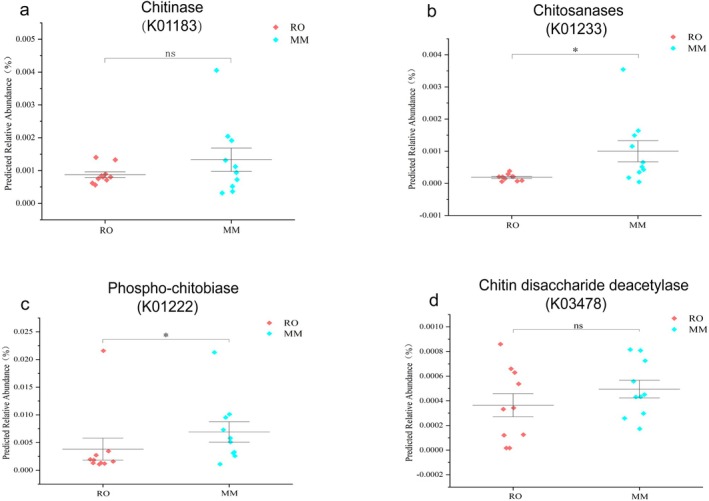
Predicted abundance of genes associated with chitin degradation. Dots represent data from individual rodents, while lines and bars indicate the means ± standard errors. Brackets denote the results of pairwise Kruskal–Wallis tests. Adjusted *p* values are represented by the following symbols: (*) indicates *p* < 0.05.

Our functional prediction analysis indicated that genes associated with chitin digestion exhibited an overall higher predicted abundance in the gut microbiota of the omnivorous MM (Figure 9). In particular, the predicted abundances of chitosanase and phospho‐chitobiase in the gut microbiota of MM were significantly greater than those observed in the herbivorous RO (Figures 9b,c). Although the predicted abundances of chitinase and chitin disaccharide deacetylase did not differ significantly between the two species, MM nonetheless exhibited a consistently greater trend than RO did (Figures 9a,d).

**FIGURE 10:**
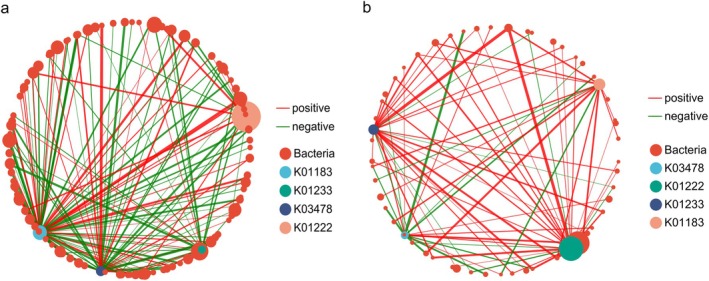
Interaction network map depicting the top 500 most abundant bacterial OTUs and chitin‐degrading genes in RO and MM. Red lines denote positive associations, whereas green lines denote negative associations. Red nodes correspond to bacterial OTUs, whereas nodes in other colors represent distinct chitin‐degrading genes. The relative abundance of chitin‐degrading genes is reflected by the size of the dots.

We conducted a Spearman correlation analysis between the 500 most abundant bacterial OTUs in the intestines of the two gerbil groups and chitin‐degrading genes. In RO, 132 OTUs were significantly correlated with chitin‐degrading genes (Table 4). Within their chitin‐degradation gene network, 63 edges represented positive correlations (42.28%), and 86 represented negative correlations (57.72%), yielding a positive‐to‐negative correlation ratio of 0.73 (Table 4, Figure 10a). This pattern suggests that antagonistic interactions predominated between chitin‐degrading genes and bacterial OTUs in RO. In MM, 57 OTUs were significantly correlated with chitin‐degrading genes (Table 4). Their gene network comprised 59 positively correlated edges (74.68%) and 20 negatively correlated edges (25.32%), with a positive‐to‐negative correlation ratio of 2.95 (Table 4, Figure 10b), indicating that synergistic interactions predominated between chitin‐degrading genes and bacterial OTUs in MM.

**TABLE 4:** Network topological characteristics.

	Chitinolytic genes network (RO)	Chitinolytic genes network (MM)
Nodes	132	57
Edges	149	79
Positive edges (%)	63 (42.28)	59 (74.68)
Negative edges (%)	86 (57.72)	20 (25.32)
Positive/Negative edges	0.733	2.95
Average degree	2.258	2.772
Diameter	6	4
Average path length	3.314	2.996
Density	0.017	0.049
Modularity	0.541	0.444

## 4 Discussion

This study primarily provides a descriptive analysis of the gut microbiomes of two gerbil species. It is important to acknowledge certain limitations. Samples were collected from a single season, which may not fully capture the potential influence of seasonal variations on diet and microbiome composition (Davis and Pineda‐Munoz 2016). Furthermore, the limited sample size (*n* = 10 per group) may reduce statistical power and constrain a comprehensive assessment of within‐population variability. Nevertheless, given that the study was conducted under natural habitat conditions and employed high‐throughput sequencing, the preliminary findings remain valuable. Our results revealed that the two rodent species exhibited markedly distinct gut microbial community structures, a pattern consistent with previous research on wild rodents (Wang et al. 2022). Moreover, we observed that diet strongly influences both the composition and functional potential of the gut microbiota, with significant differences in the predicted abundance of genes associated with specific metabolic functions among rodents fed different diets (Youngblut et al. 2019). Building upon these findings, we conducted cross‐species comparisons and discussions centered on two principal aspects: (i) the functional adaptations of the gut microbiota in herbivorous and omnivorous rodents for the digestion of complex carbohydrates and (ii) the potential link between the gut microbiota of omnivorous rodents and their capacity for chitin degradation.

### 4.1 (i) Functional Adaptations of the Gut Microbiota in Herbivorous RO/Omnivorous MM to Complex Carbohydrate Digestion

In this study, no significant difference was observed in gut microbial alpha diversity between the herbivorous RO and the omnivorous MM. Previous research has generally indicated that herbivorous mammals tend to possess higher gut microbial diversity due to the higher fiber content and greater structural complexity of their diets (Kobayashi et al. 2020). However, the findings of this study differ from this pattern: the omnivorous MM exhibited a level of gut microbial alpha diversity comparable to that of the herbivorous RO. We propose that the fact that MM also displays such relatively high diversity may be closely related to its broad dietary spectrum. Omnivorous species consume a wider range of food types, many of which contain complex components (chitin and various plant‐derived and nonplant‐derived polysaccharides) that are resistant to host digestion, thus potentially requiring a diverse microbial community to assist in their decomposition and metabolism. This elevated microbial diversity, in turn, may enhance the ability of MM to efficiently utilize its diverse food resources (Louca et al. 2018).

This study revealed that the gut of herbivorous RO not only contains a relatively high abundance of cellulose‐degrading bacterial taxa—*Muribaculaceae (*Gan et al. 2023 *)*—but also has the greatest predicted abundance of genes involved in the degradation of complex fibers, which is consistent with expectations based on its diet. Further correlation network analysis revealed that synergistic interactions predominated within the RO's cellulose‐degrading gene network (positive‐to‐negative edge ratio of 2.44), indicating substantial functional cooperation among its microbiota to support fiber breakdown. Unexpectedly, however, genes associated with monosaccharide digestion showed a higher predicted abundance in the omnivorous MM. We initially hypothesized that because cellulose must be fermented by microbes into volatile fatty acids (such as acetate and propionate) before being absorbed by the host and because monosaccharides are important intermediates in this process (Lynd et al. 2002), herbivores might also possess a high abundance of genes related to monosaccharide metabolism. In practice, the results revealed that the abundance of monosaccharide digestion genes was relatively low in RO, and its monosaccharide‐degrading gene network was dominated by antagonistic interactions (positive‐to‐negative edge ratio of 0.83), suggesting that microbial competition or inhibition may influence this metabolic pathway. One possible explanation is that monosaccharides, as transient intermediates in the cellulose fermentation process, do not accumulate or persist in large quantities in the gut, thus reducing the need to maintain a high abundance of monosaccharide digestion genes (Hungate 1944). In contrast, the monosaccharide‐degrading gene network of MM displayed more pronounced synergistic interactions (positive‐to‐negative edge ratio of 1.69), indicating a more coordinated mechanism of monosaccharide utilization within its microbiota. Although primarily omnivorous, the MM gut also contains a notable abundance of cellulose‐degrading genes and demonstrates a degree of synergistic interaction in the cellulose degradation network (positive‐to‐negative edge ratio of 1.59), which may facilitate the efficient breakdown of fibrous components when plant tissues or seed coats are consumed. From the perspective of digestive strategies—“yield maximization” versus “rate maximization”—the smaller‐bodied, rate‐maximizing MM (Choat et al. 2004) may enhance synergistic metabolic networks related to monosaccharide digestion within its microbiota to rapidly extract energy from easily degradable carbohydrates such as seed starch, thus adapting to its shorter food retention time and higher energy turnover demands.

### 4.2 (Ii) Omnivorous MM and Chitin Degradation

The gut microbiome of the omnivorous MM differs markedly from that of RO, exhibiting greater species diversity. With respect to community structure, the gut of MM is enriched with multiple functionally specialized microbial groups. Previous studies have suggested that chitin intake may induce intestinal inflammation in mammals (Jiminez et al. 2015), whereas the markedly enriched *Lachnospiraceae_NK4A136_group* in MM may contribute to the regulation of inflammation through the production of butyrate (Dou et al. 2020). As the principal energy source for intestinal epithelial cells, butyrate can reduce the pH of the intestinal lumen, inhibit pathogen colonization, and enhance intestinal epithelial barrier function by up‐regulating the transcription of the tight junction protein Claudin‐1, thereby attenuating inflammatory responses (Wang et al. 2012). Consequently, the enrichment of the *Lachnospiraceae_NK4A136_group* may represent an adaptive mechanism that mitigates chitin‐induced inflammation and supports host health. Furthermore, the pronounced enrichment of *Desulfovibrio* in the gut of MM plays a pivotal role in chitin degradation. Chitin breakdown is often accompanied by the release of H_2_; in anaerobic environments, the accumulation of H_2_ can inhibit the feedback of chitin‐fermenting enzymes, thus slowing further degradation. As a sulphate‐reducing bacterium (Vainshtein et al. 1992), *Desulfovibrio* can utilize H_2_ as an electron donor to reduce sulphate to hydrogen sulphide, effectively removing H_2_ and alleviating its inhibitory effect on the degradation process and thus markedly enhancing the metabolic activity of chitin‐degrading bacteria (Odom and Peck Jr 1984). This interspecies hydrogen transfer exemplifies a typical interaction model for the efficient degradation of complex polymers in anaerobic systems, underscoring the importance of microbial functional specialization and cooperation in host digestive adaptation. In addition, the gut of MM is enriched in genera such as *Eubacterium_siraeum_group*, *Lachnoclostridium*, and *GCA‐900066575*, which may play important roles in energy metabolism, cell signal transduction, and immune regulation (Liu et al. 2021), although their precise mechanisms warrant further investigation.

Interestingly, genes associated with chitin digestion were identified in the gut microbiomes of both gerbil species. This likely reflects the incidental ingestion of insects during their natural foraging behavior (Dickman 1999). However, the omnivorous MM displayed a markedly higher predicted abundance of these chitin‐degrading genes. These genes encode key genes that are integral to chitin breakdown, such as chitinase, chitosanases, phospho‐chitobiase, and chitin disaccharide deacetylase (Schrempf 2001). This elevated abundance of degradation‐related genes is likely to increase the metabolic capacity of MM for chitin, thus conferring a digestive adaptive advantage.

## 5 Conclusion

This study, through a comparative analysis of the gut microbiota of sympatric herbivorous RO and omnivorous MM, reveals that microecological adaptation strategies driven by dietary differentiation are a key mechanism promoting interspecific coexistence. The results showed that the omnivorous MM exhibited a level of gut microbial alpha diversity comparable to that of the herbivorous RO and was enriched in microbial taxa (*Lachnospiraceae_NK4A136_group* and *Desulfovibrio*) and functional genes associated with monosaccharide metabolism, chitin degradation, and gut health. In contrast, RO was significantly enriched in fiber‐degrading bacteria (*norank_f__Muribaculaceae*) and genes related to complex fiber degradation. Functional network analysis further revealed that the cellulose degradation gene networks in both species were predominantly synergistic, but the synergistic effect was stronger in RO (ratios of positive to negative correlation edges: 2.44: 1.59). In comparison, the monosaccharide and chitin degradation gene networks in MM both exhibited synergistic interaction patterns (ratios of positive to negative correlation edges: 1.69 and 2.95, respectively), whereas these two networks in RO were primarily antagonistic (ratios of positive to negative correlation edges: 0.831 and 0.73, respectively). This differentiation in gut microbial structure and function indicates that the gut microbiota of RO are more conducive to digesting complex plant fibers, while those of MM are better adapted for digesting starch and chitin. Such differentiation optimizes the utilization of different food resources by the two species, thereby promoting their sympatric coexistence. From the perspective of microbial functional interactions, this study deepens our understanding of the adaptive mechanisms of gut microecology in rodents with different diets and provides novel insights and an important foundation for further research on the microbial ecology of wild rodents and the mechanisms underlying sympatric species coexistence. It should be noted that 16S rRNA sequencing technology has inherent limitations in functional prediction; therefore, future studies should integrate metagenomic and metatranscriptomic approaches to further validate these findings, and readers are advised to interpret the conclusions with caution regarding these methodological constraints.

## Author Contributions

**Dongyang Chu:** data curation (equal), formal analysis (equal), validation (equal), writing – original draft (equal). **Nan Liu:** conceptualization (equal), data curation (equal), formal analysis (equal), validation (equal), writing – original draft (equal). **Qingxuan Liu:** investigation (equal). **Xin Li:** funding acquisition (equal). **Haizhou Yang:** investigation (equal), methodology (equal). **Na Zhu:** investigation (equal). **Zhiying Liu:** investigation (equal). **Rui Wang:** investigation (equal). **Shuai Yuan:** conceptualization (equal), funding acquisition (equal), project administration (equal), resources (equal), supervision (equal), writing – review and editing (equal). **Heping Fu:** funding acquisition (equal), project administration (equal), resources (equal), writing – review and editing (equal).

## Funding

This work was supported by the First Class Discipline Research Special Project of the Inner Mongolia Autonomous Region Department of Education (Grant No. YLXKZX‐NND‐005‐2), the National Key Research and Development Program of China (Grant No. 2024YFD1400505), the Inner Mongolia Natural Science Foundation (Grant No. 2023MS03025, 2025QN03135), and the 2022 Inner Mongolia Autonomous Region Youth Science and Technology Talent Development Plan (Grant No. NJYT22044).

## Ethics Statement

Our study was approved by the Ethics Committee of Inner Mongolia Agricultural University under the approval number NND2022093.

## Consent

The authors have nothing to report.

## Conflicts of Interest

The authors declare no conflicts of interest.

## Supporting information


**File S1:** Sample amplification, library preparation, and sequencing.

**File S2:** NSTI value of sample OTUs.

**File S3:** Prediction_KO.

## Data Availability

The original data are available in the NCBI data repository: https://www.ncbi.nlm.nih.gov/sra/PRJNA1274967. The original data are stored in the NCBI data repository for private peer review: https://www.ncbi.nlm.nih.gov/sra/PRJNA1274967.
